# Genetic polymorphism in the *NRF2* gene as a prognosis marker for cancer chemotherapy

**DOI:** 10.3389/fgene.2014.00383

**Published:** 2014-11-04

**Authors:** Toshihisa Ishikawa

**Affiliations:** ^1^Personalized Medicine Research Institute, NGO Personalized Medicine and Healthcare, YokohamaJapan; ^2^RIKEN Center for Life Science Technologies, YokohamaJapan

**Keywords:** drug resistance, anti-oxidant response element (ARE), ABCG2, NRF2, single nucleotide polymorphism (SNP)

## Abstract

NF-E2-related factor 2 (NRF2) is a transcription factor that controls the expression of a variety of antioxidant and detoxification genes. Accumulating evidence strongly suggests that NRF2 mediates cancer cell proliferation and drug resistance, as well. Single nucleotide polymorphism (SNP) -617C > A in the anti-oxidant response element-like loci of the human *NRF2* gene play a pivotal role in the positive feedback loop of transcriptional activation of the *NRF2* gene. Since the SNP (-617A) reportedly decreases the binding affinity to the transcription factors of NRF2/small multiple alignment format (MafK), the homozygous -617A/A allele may attenuate the positive feedback loop of transcriptional activation of the *NRF2* gene and reduce the NRF2 protein level. As the consequence, cancer cells are considered to become more sensitive to therapy and less aggressive than cancer cells harboring the -617C (WT) allele. Indeed, Japanese lung cancer patients carrying SNP homozygous alleles (*c.* -617A/A) exhibited remarkable survival over 1,700 days after surgical operation (log-rank *p* = 0.021). The genetic polymorphism in the human *NRF2* gene is considered as one of prognosis markers for cancer therapy.

## INTRODUCTION

### HISTORICAL BACKGROUND

In the field of cancer chemotherapy, it has been well documented that glutathione (GSH) plays a pivotal role in conferring cancer cells resistance to anti-tumor drugs, such as cisplatin and alkylating agents. Several lines of evidence suggest that certain multi-drug eﬄux pumps encoded by ATP-binding cassette (ABC) transporter genes are up-regulated by oxidative stress and/or chemotherapeutic agents to contribute to multi-drug resistance of cancer cells. About two decades ago, Ishikawa and Kuo first reported that many cytotoxic agents induced the expression of both γ-glutamylcysteine synthetase (γ-*GCS*) and *ABCC1 (MRP1)* genes (Ishikawa et al., 1996; Kuo et al., 1996, 1998; Gomi et al., 1997; Yamane et al., 1998). Coordinated up-regulation of both γ-*GCS* and *ABCC1* genes was found in human malignant tissues. Among 32 cases of human colorectal cancer biopsies, 78% of the cases exhibited co-elevated expression of *ABCC1* and γ-*GCS* genes in tumor samples as compared with their corresponding adjacent naïve normal samples (Kuo et al., 1996). At that time, it was speculated that a common transcriptional regulator might exist for the coordinated expression of both γ-*GCS* and *ABCC1* genes (Ishikawa et al., 1996).

### TRANSCRIPTION FACTOR NRF2 AS A MASTER SWITCH IN GENE EXPRESSION

During the past two decades, evidence has accumulated to show that one transcription factor named NF-E2-related factor 2 (NRF2) is a common redox regulator to control cellular adaptation/protection to external stimuli by inducing antioxidant and detoxification genes (Motohashi and Yamamoto, 2004; Kobayashi and Yamamoto, 2006; Nguyen et al., 2009). In fact, NRF2 is a major player in the transcriptional upregulation of many target genes in phase II drug metabolizing enzymes and certain phase III ABC transporters (ABCC2, ABCC3, and ABCG2; Adachi et al., 2007). The 5′-flanking region of many of phase II xenobiotic detoxifying genes (e.g., γ-*GCS*) contains an antioxidant response element (ARE). NRF2 directly binds to the ARE sequence in those target genes (Shen and Kong, 2009; Singh et al., 2010). Furthermore, it has recently been reported NRF2 mediates cancer cell proliferation and drug resistance (Lau et al., 2008; Hayes and McMahon, 2009; Homma et al., 2009; Taguchi et al., 2011; Sporn and Liby, 2012; Yamadori et al., 2012; Shelton and Jaiswal, 2013).

NF-E2-related factor 2 is a “cap‘n’collar” basic region-leucine zipper (CNC-bZip) transcription factor involved in the induction of ARE-regulated genes (Moi et al., 1994; Motohashi and Yamamoto, 2004; Kobayashi and Yamamoto, 2006; Lau et al., 2008; Hayes and McMahon, 2009; Homma et al., 2009; Nguyen et al., 2009; Taguchi et al., 2011; Sporn and Liby, 2012; Yamadori et al., 2012). Under non-stressed conditions, NRF2 protein is associated with Kelch-like ECH associating protein 1 (*KEAP1*; Itoh et al., 1999) that negatively regulate NRF2 by retrieving the NRF2 protein in the cytoplasmic compartment. However, oxidative stress and/or electrophilic attack modifies the KEAP1 protein, which leads to dissociation of NRF2 from KEAP1. The NRF2 protein, thus released, is subsequently translocated into the nucleus. Coupling with small multiple alignment format (MAF) sequences, NRF2 binds to ARE sequences (Itoh et al., 1995). Many genes encoding detoxifying and antioxidant enzymes have been found to be regulated by the NRF2 protein in this manner (Itoh et al., 1995; Ishii et al., 2000; Ramos-Gomez et al., 2001; Motohashi and Yamamoto, 2004; Cho et al., 2005; Kobayashi and Yamamoto, 2006; Nguyen et al., 2009). Recent studies, on the other hand, have shown that NRF2 contributes to cancer cell proliferation, drug resistance, and metabolic re-programming, as well (Kwak et al., 2002; Lau et al., 2008; Hayes and McMahon, 2009; Homma et al., 2009; Taguchi et al., 2011; Mitsuishi et al., 2012; Sporn and Liby, 2012; Yamadori et al., 2012). In this context, the *NRF2* gene is regarded as a “double-edged sword,” namely, protection of normal cells and progression of cancer malignancy.

## GENETIC POLYMORPHYSMS IN THE *NRF2* GENE

**FIGURE 1:**
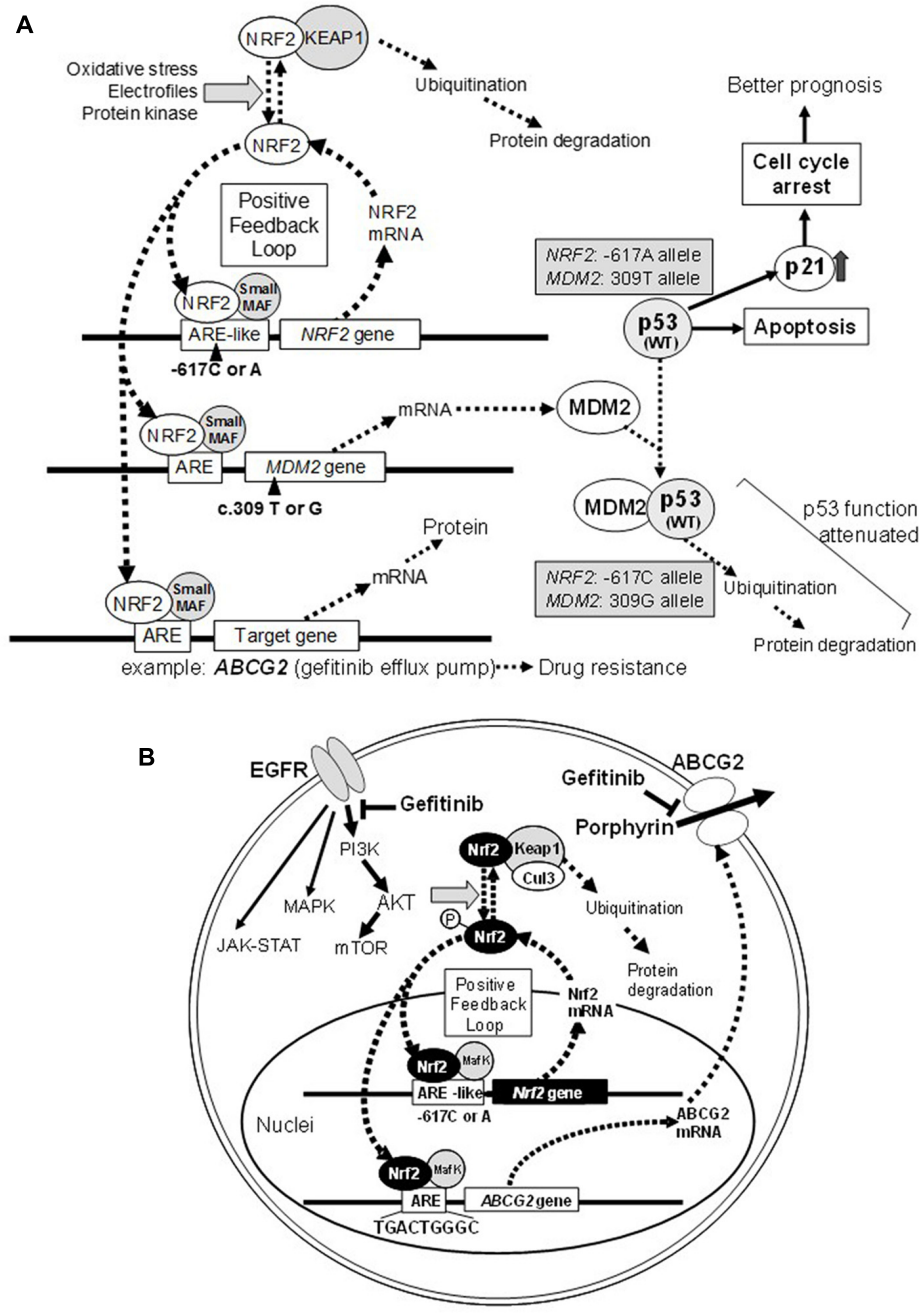
**Schematic illustrations showing the effect of *NRF2* SNP -617C > A and *MDM2* SNP *c.* 309 T > G on the p53-mediated suppression of cancer cell proliferation **(A)** and ABCG2-mediated drug resistance to gefitinib **(B)**.** Refer to Okano et al. (2013) for more details.

Yamamoto et al. (2004) first reported three single nucleotide polymorphisms (SNPs; -653A > G, -651G > A, and -617C > A) and one triplet repeat polymorphism in the regulatory region of the human *NRF2* gene. The physiological significance of these SNPs was not known at that time. Three years later, Marzec et al. (2007) reported the impact of those SNPs on the regulation of *NRF2* gene expression. In fact, the -617C > A SNP significantly affected basal NRF2 protein levels *in vitro* (Marzec et al., 2007). Moreover, the SNP -617C > A was found to be associated with a higher risk of oxidant-induced acute lung injury in humans (Marzec et al., 2007). These findings suggest that the SNP (-617C > A) in the ARE-like loci of the human *NRF2* gene is important for self-induction of the *NRF2* gene (Okano et al., 2013); refer to schematic illustrations in **Figure 1**.

## SNP (–617C > A) IN THE *NRF2* GENE AS A BIOMARKER FOR PROGNOSIS OF LUNG CANCER

NF-E2-related factor 2 plays a pivotal role in protecting normal cells from external toxic challenges and oxidative stress, whereas it can modulate the cancer phenotype (**Figure 1A**) and also endow cancer cells resistance to anticancer drugs (**Figure 1B**). NRF2 activation appears to be associated with the emergence of cancer resistance to various anticancer drugs by transcriptionally activating a battery of self-defense genes. Indeed, NRF2 can induction the expression of γ-*GCS* and *ABCC1* genes involved cancer cell resistance to cisplatin and alkylating agents (Ishikawa et al., 1996; Adachi et al., 2007). In addition, ABCG2 is known to mediate the eﬄux of gefitinib (Iressa) from cancer cells (Saito et al., 2006), and its expression is regulated by NRF2 (Singh et al., 2010) and the EGFR-tyrosine kinase cascade (Meyer zu Schwabedissen et al., 2006; Huang et al., 2011; **Figure 1B**).

**FIGURE 2:**
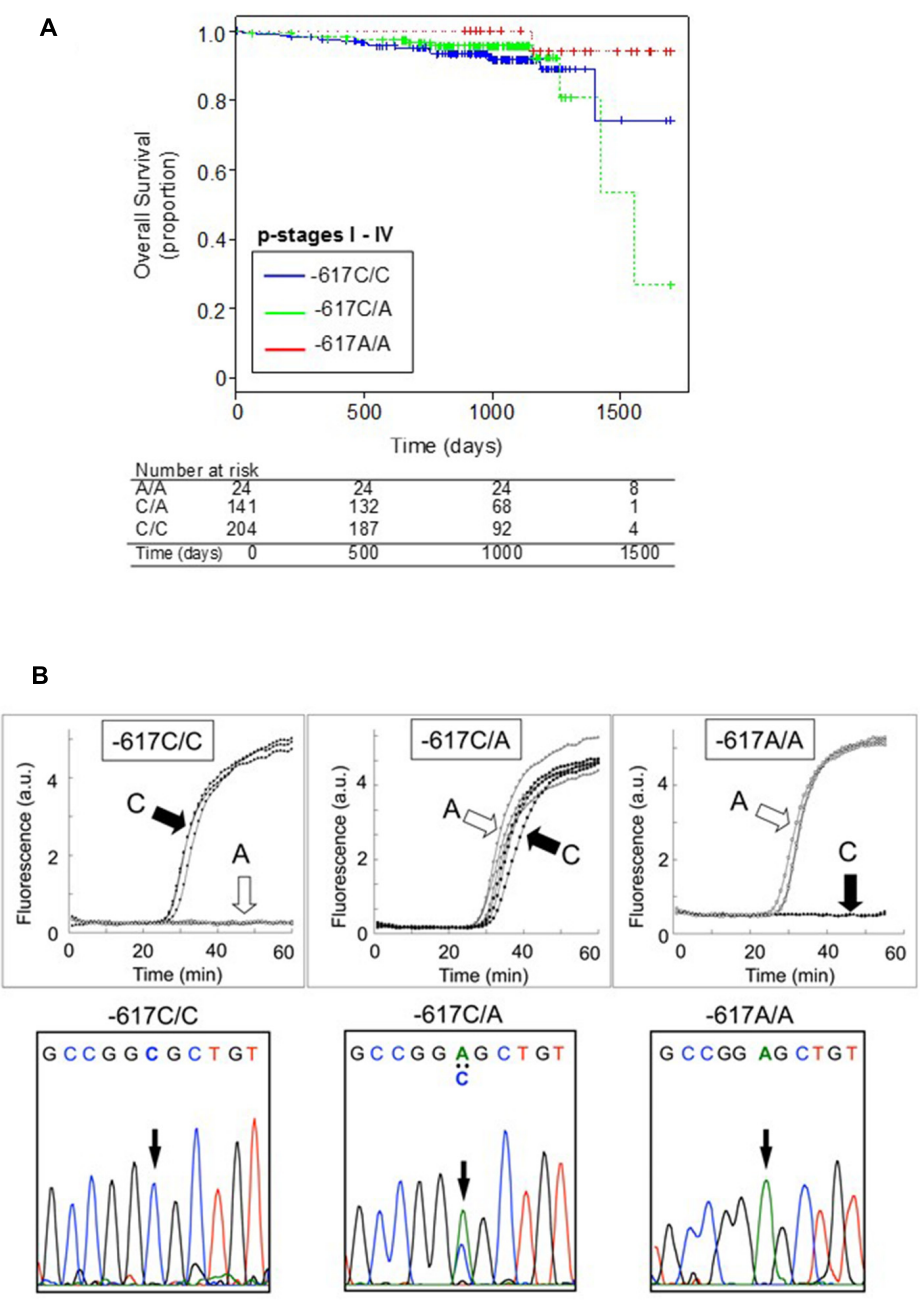
**Kaplan-Meier plots showing the overall survival of patients harboring the WT homozygote (-617C/C), WT/SNP heterozygote (-617C/A), or SNP homozygote (-617A/A) in the *NRF2* gene **(A)** and genotyping of *NRF2* SNP -617C > A by the rapid SNP-detection method (B, upper panels) and DNA sequence analysis (B, lower panels).** Data from Okano et al. (2013).

Single nucleotide polymorphism -617C > A could affect the positive feedback loop of transcriptional activation of the *NRF2* gene, and thereby it can regulate the NRF2 protein level. It is proposed that the homozygote -617A/A significantly attenuates the positive feedback loop of transcriptional activation of the *NRF2* gene. Interestingly, Asians, including Japanese, have higher frequencies of the -617A allele as compared with African–Americans and Caucasians (Okano et al., 2013). As demonstrated in **Figure 2A**, Japanese lung cancer patients carrying SNP homozygous alleles (*c.*-617A/A) exhibited remarkable survival over 1,700 days after surgical operation (log-rank *p* = 0.021). This SNP is considered as a new biomarker for prognosis of lung cancer in Japanese population, and a hypothetical molecular mechanism has been proposed (Okano et al., 2013).

## SOMATIC MUTATIONS IN *NRF2* AND *KEAP1* GENES

The genetic polymorphisms are the “intrinsic” mechanism, whereas the mutations are the “acquired” mechanism in cancer cells. Hitherto, several mutations in the *NRF2* and *KEAP1* genes have been reported in carcinomas of the lung (Sporn and Liby, 2012), liver (Yoo et al., 2012), stomach (Yoo et al., 2012), and breast (Sjöblom et al., 2006). Abnormalities in NRF2 activity were correlated with poor prognosis in terms of either recurrence-free or overall 5-year survival. Increased expression of NRF2 protein and decreased expression of KEAP1 protein were often observed as common abnormalities in non-small cell lung cancer (NSCLC), being associated with poor prognosis (Solis et al., 2010).

## FUTURE PERSPECTIVES

Identification and validation of biomarkers for personalized cancer therapy is one of the challenges in cancer management. To practically realize personalized medicine, development of cost-effective methods is required. Furthermore, genetic information in each patient’s record should be timely provided for individualized cancer treatment. In this regards, we have recently developed a rapid isothermal method to detect genetic polymorphisms in the *NRF2* gene and correlated the genotyping data with the survival of patients who had primary lung cancer (Okano et al., 2013). By means of the new method (Ishikawa and Hayashizaki, 2013), we could detect the SNP -617C > A in the *NRF2* gene within 30 to 45 min without DNA isolation and PCR amplification (**Figure 2B**). Such genotyping methods would provide a simple and practical tool for personalized cancer therapy and assessment of prognosis.

## Conflict of Interest Statement

The author declares that the research was conducted in the absence of any commercial or financial relationships that could be construed as a potential conflict of interest.
